# Prevalence and characteristics of Hymenoptera venom allergy in urban school children aged 6 to 18 years living in Trabzon

**DOI:** 10.3906/sag-2009-86

**Published:** 2021-06-28

**Authors:** Özge ÖZİŞ BABA, Gülay KAYA, Mehtap HAKTANIR ABUL, Neşe KAKLIKKAYA, Murat ÇAKIR, Fazıl ORHAN

**Affiliations:** 1 Department of Pediatrics, Faculty of Medicine, Karadeniz Technical University, Trabzon Turkey; 2 Department of Pediatric Allergy and Immunology, Faculty of Medicine, Karadeniz Technical University, Trabzon Turkey; 3 Department of Microbiology, Faculty of Medicine, Karadeniz Technical University, Trabzon Turkey

**Keywords:** Hymenoptera venom, prevalence, children

## Abstract

**Background/aim:**

Hymenoptera venom allergy is one of the leading causes of systemic allergic reactions in both adults and children. The present study was conducted to evaluate the prevalence and characteristics of Hymenoptera venom allergy in urban school children aged 6 to 18 years living in Trabzon.

**Materials and methods:**

In this cross-sectional, two-level survey study, children were recruited using random sampling of public primary and secondary schools. Firstly, parents were asked about their child’s age and sex and whether their child had ever been stung by any kind of bee. When they responded “yes” to the last question, they attended a face-to-face interview at the outpatient clinic for the second part of the survey, which included information about history of insect stings and the presence of atopic diseases.

**Results:**

Of 17,000 children, 7904 (46.5%; 3718 males, 47.0%) returned the first-level questionnaire. A total of 4312 (54.5%) were stung at least once in their lifetime. Males had a significantly higher risk of being stung (59.4%, odds ratio: 1.44, 95% confidence interval: 1.32–1.58, p < 0.0001). The second-level questionnaire was completed for 545 (12.6%) of the children. Of 950 stings reported in 545 children, 5.2% were large local reactions (LLRs), 1.9% were generalized cutaneous reactions (GCRs), and 1.3% were systemic reactions (SRs). The stinging insect was *Apis mellifera* and *Vespula* in 66.2% and 33.8% of stings, respectively (p < 0.001).

**Conclusion:**

Hymenoptera stings are common in urban school children living in Trabzon. The most common type of allergic reaction is LLR and the most reported stinging insect is *Apis mellifera* .

## 1. Introduction

Hymenoptera venom allergy is one of the leading causes of systemic allergic reactions (i.e., anaphylaxis) both in adults and children [1,2]. It is reported that 56.6% to 94.5% of interviewees remember being stung by a Hymenoptera insect at least once in their lives [3]. In adults, the prevalence of systemic reactions (SRs) ranges from 0.5% to 3.3% in the USA and 0.3% to 7.5% in Europe [4,5]. Though the prevalence of SRs to Hymenoptera stings was reported as 0.35% to 0.4% in previous questionnaire-based studies in selected pediatric groups (i.e., 11 to 16-year old summer camp attendees in the USA) [6,7], more recent studies worldwide report a prevalence ranging from 0.34% to 6.5% in school children [8–10]. In Turkey, an estimated 61.6% of children aged 12 to 14 years have been stung at least once and 0.8% have experienced severe SRs [11].

The aim of this study was to evaluate the prevalence and characteristics of Hymenoptera venom allergy in urban school children between 6 and 18 years of age living in Trabzon.

## 2. Materials and methods

### 2.1. Study population 

This cross sectional study was performed in 2016 in the urban center of Trabzon, a city located in the eastern Black Sea region of Turkey. 

Children were recruited by random sampling of public primary and secondary schools so as to represent all ages, there were comparable numbers of boys and girls at all ages. Based on previous studies conducted both in Turkey and worldwide [8 **–** 11] reporting a prevalence of 0.34% to 6.5%, we estimated the prevalence of SR could be 1.0%. At the time of the study, the total population of urban school children between the ages of 6 and 18 years in the city center of Trabzon was approximately 62,400. Therefore, our target study population was 8500 and we attempted to recruit 17,000 children to allow for up to 50% nonparticipation.

This study was carried out in two phases. In phase 1, questionnaires were distributed to the children and collected approximately 1 week later. In phase 2, the parents who agreed to participate were interviewed at the outpatient clinic using a more detailed questionnaire. A flow chart of the study is shown in Figure.

**Figure:**
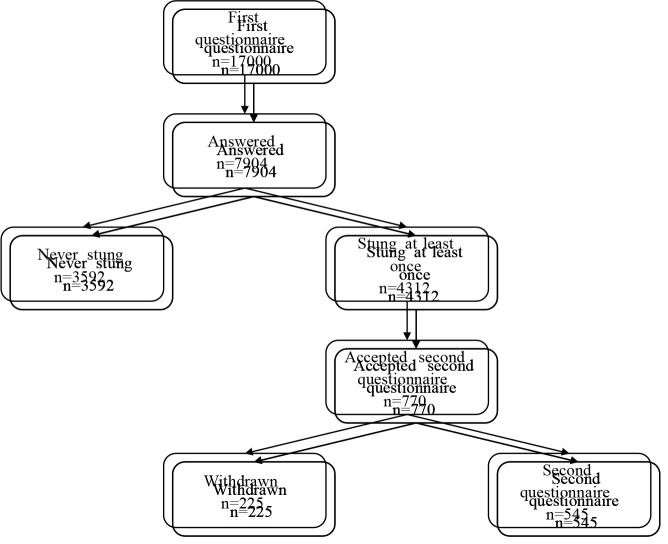
Flow chart of the study.

The study was approved by the Karadeniz Technical University Ethics Committee.

### 2.2. Questionnaire

The two-level questionnaire to be completed by the parents/caregiver and child was validated in a sample of 20 subjects through in-depth interviews. In the first level, the parent was asked to indicate their child’s age and sex, and whether their child had ever been stung by any kind of bee. If the answer was yes, the respondents were then asked whether they would attend the outpatient clinic to complete the second level of the questionnaire in person. 

In the second level, a face-to-face questionnaire was administered at the pediatric allergy outpatient clinic of Karadeniz Technical University Hospital. The participants were asked to indicate the number and first/last time of stings, and whether they had ever experienced one or more of the following reactions: a normal sting reaction; a large local reaction (LLR), defined as an induration contiguous to the sting site that was estimated to be equal to or greater than 5 cm and persisted for more than 2 days; generalized cutaneous reaction (GCR), defined as a reaction occurring within 1 h of the sting and consisting of an isolated cutaneous manifestation such as generalized hives or angioedema; or a systemic reaction (SR), defined as a reaction occurring within 1 h of the sting and including symptoms involving at least two of the following systems: cutaneous (eruption, itching, rash, and swelling), respiratory (nasal itching, secretion, blockage, sneezing; ocular redness, itching, secretion; stridor, difﬁculty in swallowing, cough, wheezing, and shortness of breath), gastrointestinal (stomachache, nausea, vomiting, and diarrhea), cardiovascular (palpitations/tachycardia, hypotension), and other (sweating, pallor, fainting, and loss of consciousness). Respondents were asked to specify the type of bee (honeybee or wasp) that caused the reaction. Information on personal and familial atopic diseases such as asthma, allergic rhinitis, atopic dermatitis, and food allergies were also obtained. All items required a yes/no response except the question about number of stings, which required a numerical response.

### 2.3. Statistics

All analyses were performed using SPSS statistical software v.16.0 (SPSS Inc., Chicago, IL, USA). Descriptive analysis was used to characterize the study population. Results were expressed as the mean ± standard deviation (SD) or percentages of the responses to each question. The children were grouped according to sex and by reaction type, as the normal reaction (NR) group and pathologic reaction (PR) group (LLR and/or GCR and/or SR). Comparisons were also made within the PR group when appropriate. Response frequency was compared using the Pearson chi-square (C2) test (with the Yates correction when applied) or Fisher’s exact test when required. The odds ratios and 95% confidence interval (CI) were calculated and p < 0.05 was considered statistically significant.

## 3. Results

Of the 17,000 children who received questionnaires, 7904 (46.5%; 3718 males, 47.0%) returned the first-level questionnaire and were included in the study. Therefore, we achieved 93.0% of the target population size of 8500. A total of 4312 children (54.5% of 7904; 2208 males, 51.2%, p > 0.05) reported being stung at least once in their lifetime. Males were found to have a significantly higher risk of being stung (2208/3718, 59.4%, OR 1.44, 95% CI 1.32–1.58, p < 0.0001) (Table 1).

**Table 1:** Prevalence and adjusted odds ratios (OR) with 95% confidence intervals (CI) for sting numbers according to sex.

	Ever stung, n (%)	1 sting, n (%)	2–5 stings, n (%)	> 5 stings, n (%)
Total (n = 7094)	4312 (54.5)			
Male (n = 3718)	2208 (59.4)*,a			
Female (n = 4186)	2014 (48.1)			
Second-level questionnaire (n = 545)				
Male, n (%)		159 (47.9)	106 (52.7)	8 (66.7)
Female, n (%)		173 (52.1)	95 (47.3)	4 (33.3)
OR (95% CI)		0.79 (0.56–1.12)	1.18 (0.83–1.67)	2.02 (0.60–6.79)
p		0.19a	0.34a	0.25b

*p < 0.0001aPearson chi-square testbFisher’s exact test

Of the 4312 respondents to the first questionnaire, 770 (17.8%) agreed to participate in the second level of the study. However, 225 of these participants withdrew from the study for various reasons such as the study period coinciding with academic examination periods. Therefore, the face-to-face detailed questionnaire was administered to 545 children (12.6% of total), which comprised the final study population.

The mean ± SD age of the final study population was 10.9 ± 2.8 years (273 males, 50.1%, p = 0.95). The mean ± SD age at first sting was 6.3 ± 2.8 years. A total of 950 stings were reported in 545 children, with 332 children stung only 1 time (60.9%; 159 males, 47.9%, OR 0.79, 95% CI 0.56 **–** 1.12, p = 0.19), 201 stung between 2 and 5 times (36.9%; 106 males, 52.7%, OR 1.18, 95% CI 0.83 **–** 1.67, p = 0.34), and 12 stung more than 5 times (2.2%; 8 males, 66.7%, OR 2.02, 95% CI 0.60–6.79, p = 0.25) (Table 1). The stinging insect was *Apis mellifera* in 629 (66.2%) and *Vespula* spp. in 321 (33.8%) of the stings (p < 0.001). 

Of the 950 stings in 545 children, there were 874 normal reactions (92.0%) in 497 children (91.2%), 49 LLRs (5.2%) in 35 children (6.4%), 18 GCRs (1.9%) in 7 children (1.3%), and 9 SRs (0.9%) in 6 children (1.1%). Boys with LLR and GCR had ORs of 1.74 (95% CI 0.86 **–** 3.54, p = 0.12) and 1.33 (95% CI 0.29 **–** 5.99, p = 0.70), respectively, while girls with SR had an OR of 2.02 (95% CI 0.36–11.13, p = 0.42) (Table 2). The mean ± SD ages of children with LLR, GCR, and SR were 9.8 ± 2.6, 10.3 ± 3.0, and 11.5 ± 2.8 years, respectively (p > 0.05). Among the children with multiple stings, 1 boy had an LLR followed by a GCR. No SRs were reported in any subsequent stings of children with LLR or GCR. Of the 6 children with SR, all had multiple stings before their first SR. Four children (3 girls, 1 boy) reported having a single SR, 1 girl had 2 SRs, and 1 boy had 3 SRs. 

**Table 2:** Adjusted odds ratios (OR) with 95% confidence intervals (CI) for sting reaction types.

	LLR	GCR	SR
Sex			
Male, n (%)	22 (62.8)	4 (57.1)	2 (33.3)
Female, n (%)	13 (37.2)	3 (42.9)	4 (66.7)
	1.74 (0.86–3.54)	1.33 (0.29–5.99)	2.02 (0.36–11.13)
p	0.12a	0.70b	0.42b
Hymenoptera type			
Apis mellifera, n (%)	27 (4.3)	11 (1.7)	7 (1.1)
Vespula, n (%)	22 (6.9)	7 (2.2)	2 (0.6)
	1.64 (0.91–2.92)	1.25 (0.48–3.26)	1.79 (0.37–8.69)
p	0.09a	0.64a	0.46b
Atopy, n (%)	5 (14.2)	2 (28.6)	2 (33.3)
	0.37 (0.08–1.69)	1.94 (0.31–12.11)	2.50 (0.38–16.40)
p	0.20b	0.47b	0.33b

aPearson chi-square testbFisher’s exact test

Children stung by *Vespula* spp. had an OR of 1.64 (95% CI 0.91–2.92, p = 0.09) and 1.25 (95% CI 0.48 **–** 3.26, p = 0.64) for LLR and GCR, respectively, while children stung by *Apis mellifera* had an OR of 1.79 (95% CI 0.37 **–** 8.69, p = 0.46) for SR (Table 2). 

Atopic diseases were reported in 123 (22.6%) of the 545 children. These included allergic rhinitis in 66 (53.7%), asthma in 59 (41.5%), atopic dermatitis in 27 (21.9%), and food allergy in 19 (15.4%) of the children. There were no significant differences in rates of reported atopic disease between the NR and PR groups (114/497, 22.9% vs. 9/48, 18.7%; p = 0.50) or within the PR group (LLR: 5/35, 14.2%, OR 0.37, 95% CI 0.08 **–** 1.69 vs. GCR: 2/7, 28.6%, OR 1.94, 95% CI 0.31 **–** 12.11 vs. SR: 2/6, 33.3%, OR 2.50, 95% CI 0.38 **–** 16.40; p = 0.57) (Table 2). Of those in the PR group who reported an atopic disease, 7 (77.7%) had asthma, 5 (55.5%) had allergic rhinitis, and 2 (22.2%) had atopic dermatitis. A food allergy was not reported by any of the children in the PR group.

A family history of venom allergy was reported by 31 children (5.7%). The prevalence of family history of venom allergy did not differ significantly between the NR (28/497, 5.6%) and PR (3/48, 6.3%) groups (p = 0.86).

## 4. Discussion

In this study, we found that more than half (54.5%) of the urban school children aged 6 to 18 years had been stung at least once in their lifetime. 

As this study included urban school children with history of bee sting, we compared our results primarily with pediatric studies that involved urban children and analyzed stung children. The prevalence of lifetime Hymenoptera stings in children differs among studies. While a rate of 33.0% was reported in urban Irish children from Dublin [10], this figure was 54.2% in urban Israeli children [9], and 59.2% and 60.8% in urban Turkish children living in Izmir [11] and Ankara [12], respectively. Differences in climatic conditions may be cited as an explanation for this wide regional variation in prevalence of lifetime Hymenoptera stings in children. However, this cannot be the case, at least for Dublin and Trabzon, as they share similar weather conditions (1450 vs. 1660 sunny h/year, and mean rainfall of 750 vs. 810 mm/year, respectively) [www.currentresults.com]. Moreover, although the prevalence of Hymenoptera stings among urban school children in Israel was similar to our result, the region has nearly twice the sunny hours (3300 h/year) compared to Trabzon. [www.tel-aviv.climatemps.com] Beekeeping is a common activity in Trabzon and there are many hives, even on the roofs of buildings in the city center, which may contribute to encounters between children and bees.

The results of the present study show that the number and prevalence of Hymenoptera stings are related to sex, as reported in previous studies [9–11]. Although not significant, lower sting numbers and prevalence were observed in girls and higher numbers and prevalence in boys. In the whole study group, however, boys were stung significantly more than girls. This is probably because the boys spent more time outside than girls, which makes them more vulnerable to exposure to stinging insects.

A significant majority of the stings in our study group were attributed to *Apis mellifera* . This is consistent with previous studies conducted both in children [12,13] and adults [14] in Turkey. The species of Hymenoptera responsible for stings may differ according to region. In the Mediterranean region, *Vespula* and *Polistes* are more frequent than *Apis mellifera* stings, whereas *Vespula* and *Apis mellifera* are more prevalent in central and northern Europe [15]. Climatic and geographical characteristics as well as beekeeping activities may influence such differences. Turkey is the world’s second leading country in beekeeping, [https://arastirma.tarimorman.gov.tr/tepge/Belgeler/PDF]which may in part cause the higher exposure to *Apis mellifera* .

Compared to the present study, the rates of LLR and GCR were 4- to 5 fold higher in Izmir and Israeli studies [9,11]. The prevalence of SR found in our study was similar to rates reported in Ireland [10] and Izmir [11], but was approximately 5-fold higher in the Israeli study [9]. Differences in numbers and age groups between the study populations might have caused these wide variations. 

In our study, the mean age in the pathologic reaction type groups increased as reaction severity increased. This was expected, as the prevalence of SR is known to be higher in adults than children [15].

Although not statistically significant, we noted that SRs were more common with *Apis mellifera* stings. This may be related to venom dose per sting, which is known to vary from species to species. *Apis mellifera* releases an average of 50 µg to 140 µg of venom protein per sting [16,17], whereas *Vespula* spp. generally inject 1.7 µg to 3.1 µg of venom [16].

Although it was previously reported that atopy does not predispose patients to Hymenoptera allergy [18], recent studies suggest that allergic diseases are associated with a higher rate and greater severity of allergic reactions to insect stings in children [10,11,19]. Graif et al. [19] identified asthma, allergic rhinitis, and atopic eczema as significant risk factors for allergic reactions to Hymenoptera stings of any severity. Arıkan-Ayyıldız et al. [11] reported that asthma was a significant risk factor for LLR and SR, and allergic rhinitis for LLR and GCR. In Ireland, however, asthma was found to be a significant risk factor only for SR, whereas allergic rhinitis and eczema were for GCR [10]. In the present study, we detected no significant differences in atopic disease between the NR and PR groups (asthma was more common in the PR group, but the difference was not significant). We also noted an increase in the prevalence of atopic disease as the severity of the sting reaction increased. However, because there were too few atopic children in the PR group, we were unable to analyze the significance of atopic diseases in the different sting reaction groups.

A limitation of our study is that our sample size was smaller than other studies. This was due to the fact that the survey consisted of a two-level questionnaire and that fewer children participated in the second level compared to the first level. We wanted to conduct the more detailed questionnaire in face-to-face interviews because we included a wide age group in the study. It has been suggested that children tend to answer questionnaires themselves and often exaggerate or hide their disease-related status [11], which results in an over- or underestimation in the results. Though our goal was to minimize this phenomenon via face-to-face interviews with the parents in the second stage of the study, we think that the two-level structure of the survey necessitated a trade-off between lower numerical strength and more reliable data.

In conclusion, the present study revealed that Hymenoptera stings are common among urban school children living in Trabzon, Turkey. LLRs were the most common type of allergic reaction and severe SRs were rare, in keeping with other pediatric studies except in Israel. *Apis mellifera* was the leading cause of stings both in the overall population and in subjects with SRs.

## Funding source

Karadeniz Technical University - Bilimsel Araştırma Projeleri Birimi (funding code: TTU-2015-5166).

## Ethical approval

This study is approved by Karadeniz Technical University Ethics Committee. 
